# Quercetin Inhibits Gastric Cancer Progression via Suppression of HOTAIR/mir-217/GPC5 Axis

**DOI:** 10.5812/ijpr-165480

**Published:** 2025-11-17

**Authors:** Zhuqing Qiu, Chonglei Qu, Xiaoying Wu

**Affiliations:** 1 Department of Gastroenterology, Daping Hospital of Army Medical University, Chongqing, China; 2 Department of Emergency, Chongqing Hospital of Jiangsu Province Hospital, Yantai, China; 3 Department of Gastroenterology, Chongqing Hospital of Jiangsu Province Hospital, Chongqing, China

**Keywords:** Gastric Cancer, Quercetin, Phytochemical, HOTAIR

## Abstract

**Background:**

Long noncoding RNA (lncRNA) hox transcript antisense intergenic RNA (HOTAIR) is implicated in the progression of gastric cancer (GC) by promoting the microRNA-217 (miR-217)-glypican-5 (GPC5) axis. Quercetin (QCT), a well-known flavonoid, has demonstrated anticancer effects against various malignancies, including GC. However, the impact of QCT on HOTAIR expression and its downstream mediators remains unclear.

**Objectives:**

This study aimed to elucidate the antitumor mechanisms of QCT and its regulatory effects on the HOTAIR/miR-217/GPC5 axis in AGS and MKN-45 GC cell lines.

**Methods:**

Cellular viability, apoptosis, cell cycle progression, invasion, and oxidative stress markers were assessed using the MTT assay, annexin V-FITC/PI staining, real-time quantitative polymerase chain reaction (RT-qPCR), enzyme-linked immunosorbent assay (ELISA), and spectrophotometry. Expression levels of HOTAIR, miR-217, and GPC5 were quantified.

**Results:**

The QCT significantly downregulated HOTAIR and GPC5 while upregulating miR-217 in both cell lines (P < 0.001). The QCT induced dose-dependent apoptosis and cell cycle arrest, and reduced invasion through upregulation of TP53/PTEN (P < 0.05). Oxidative stress modulation displayed lineage-specific differences, with a marked reduction in malondialdehyde (MDA) in MKN-45 cells (P = 0.013). AGS cells exhibited greater sensitivity to QCT than MKN-45 cells.

**Conclusions:**

These findings highlight QCT’s ability to inhibit GC progression via the HOTAIR/miR-217/GPC5 axis, with molecular heterogeneity influencing therapeutic response. The QCT emerges as a promising candidate for further investigation as a multifaceted agent against GC, though validation in preclinical models is necessary.

## 1. Background

Gastric cancer (GC) is a primary malignant neoplasm arising from the gastric epithelium (1). Although incidence and mortality rates have gradually declined in recent years, GC remains among the most prevalent malignancies worldwide, ranking as the fifth most common cancer and the fourth leading cause of cancer-related deaths globally (2, 3). The clinical management of GC is complicated by intra-tumor and inter-tumor heterogeneity (4).

Hox transcript antisense intergenic RNA (HOTAIR) is a long noncoding RNA (lncRNA) involved in multiple facets of carcinogenesis, including cellular proliferation, migration, apoptosis, metastasis, and drug resistance (5, 6). Elevated HOTAIR expression has been consistently associated with accelerated cancer progression and poor prognosis (7). Furthermore, HOTAIR expression correlates with resistance to chemotherapeutic agents such as cisplatin, oxaliplatin, and trastuzumab (8, 9).

MicroRNA-217 (miR-217) is a key microRNA implicated in the progression of various cancers through regulation of cellular proliferation, migration, and epithelial-to-mesenchymal transition (10-12). Glypican-5 (GPC5), a member of the heparan sulfate proteoglycan family, plays crucial roles in cellular regulation and development (13). Notably, HOTAIR overexpression significantly impacts GPC5 expression by modulating miR-217, leading to increased GPC5 levels (14).

Quercetin (QCT), a ubiquitous flavonoid abundant in plant-based foods, exhibits a diverse range of health-promoting properties. It is extensively studied for its impact on gastrointestinal carcinogenesis, with evidence suggesting that its effects extend beyond anti-inflammatory action to modulation of cellular migration, apoptosis, and angiogenesis (15).

## 2. Objectives

Given the limited understanding of how QCT influences HOTAIR expression and its downstream effects, this study aimed to investigate the anticancer potential of QCT in GC cells and clarify the role of the HOTAIR/miR-217/GPC5 axis in this context.

## 3. Methods

### 3.1. Cell Culture and Treatment Protocol

Human gastric adenocarcinoma cell lines (AGS [American Type Culture Collection (ATCC)^ ®^] CRL-1739^™^), MKN-45 (ATCC^®^ CRL-5979^™^)] and normal gastric epithelial cells [GES-1 (ATCC^®^ CRL-5978^™^)] were obtained from the ATCC. AGS and MKN-45 cell lines were selected as they represent different molecular subtypes of GC: AGS cells are TP53 wild-type and derived from a primary gastric adenocarcinoma, whereas MKN-45 cells harbor a TP53 mutation and are derived from a lymph node metastasis. This selection enhances the translational relevance by enabling investigation across distinct genetic backgrounds. The GES-1 cell line served as a normal control to evaluate QCT’s selective toxicity.

Cells were seeded in 96-well plates at a density of 1.0 × 10^4^ cells per well. The culture medium consisted of Dulbecco’s Modified Eagle Medium (DMEM; Gibco, #11965092) with 10% fetal bovine serum (FBS; Gibco, #10270106), 1% penicillin-streptomycin (Gibco, #15140122; final concentration 100 U/mL penicillin and 100 µg/mL streptomycin), and 0.1% amphotericin B (Sigma-Aldrich, #A2942; 0.25 µg/mL). After 24 hours of incubation, cells were treated with various concentrations of QCT (Sigma-Aldrich, #Q4951, purity ≥ 95%) dissolved in ethanol (0.3% v/v). Treatments lasted for an additional 24, 48, and 72 hours. All experiments included three biological replicates, with technical triplicates for quantitative polymerase chain reaction (qPCR) and enzyme-linked immunosorbent assay (ELISA) assays.

### 3.2. MTT Assay and Apoptosis Assessment by Annexin V-FITC/PI

Cellular viability was evaluated using the MTT assay. MTT reagent (Sigma-Aldrich, #M5655) was added to each well at a final concentration of 0.5 mg/mL and incubated for 4 hours at 37°C. Formazan crystals were dissolved in 100 µL of dimethyl sulfoxide (DMSO), and absorbance was measured at 570 nm using a microplate reader (BioTek Instruments). IC_50_ values were calculated from dose-response curves using a four-parameter logistic (4PL) nonlinear regression model in GraphPad Prism (Version 9.0.0).

Apoptosis was assessed using the annexin V-FITC/PI apoptosis detection kit (BD Biosciences, USA, #556547). After treatment, cells were harvested, washed twice with ice-cold phosphate-buffered saline (PBS), and resuspended in 500 µL of 1X annexin V binding buffer. Then, 5 µL of annexin V-FITC and 5 µL of propidium iodide were added, and samples were incubated in the dark for 15 minutes at room temperature. Apoptotic populations were quantified using a BD FACSCalibur flow cytometer (BD Biosciences, USA) and analyzed with FlowJo (Version 10.8.1).

### 3.3. RNA Extraction and Real-time Quantitative Polymerase Chain Reaction

Total RNA was extracted using the PureLink RNA Mini Kit (Thermo Fisher Scientific, USA, #12183018A), following the manufacturer’s instructions. RNA quality and quantity were assessed using a Biotek Nanodrop system. Complementary DNA (cDNA) synthesis for HOTAIR and GPC5 was performed using the High-Capacity cDNA Reverse Transcription Kit (Thermo Fisher Scientific, USA, #4368814). For miR-217, cDNA synthesis utilized the TaqMan MicroRNA Reverse Transcription Kit (Applied Biosystems, Foster city, CA, USA). Glyceraldehyde-3-phosphate dehydrogenase (GAPDH) and small nuclear RNA U6 served as endogenous controls for HOTAIR/GPC5 and miR-217 normalization, respectively, based on their stable expression in GC cell lines, even under phytochemical treatment (16). Relative gene expression was calculated using the 2^-ΔΔCT^ method (Table S1, in the Supplementary File).

### 3.4. Enzyme-Linked Immunosorbent Assay

The ELISA was conducted as per the manufacturers’ instructions. Kits for GPC5 (Abcam, #ab313967), BCL-2 (Abcam, #ab272102), CASP-3 (Abcam, #ab220655), TP53 (MyBioSource, #MBS175896), PTEN (MyBioSource, #MBS761600), cyclin A2 (Abbexa, #abx259510), and cyclin D1 (Abcam, #ab214571) were used. Cell lysates were diluted 1:10 in diluent buffer. Absorbance was read at 450 nm, with a correction at 570 nm, using a BioTek microplate reader.

### 3.5. Assessment of Oxidative Stress Markers

Cell extracts were prepared by lysing cells in RIPA buffer and centrifuging at 12,000 × g for 15 minutes at 4°C. Catalase and superoxide dismutase (SOD) activities were measured with commercial kits (Catalase: Cayman Chemical, #707002; SOD: Cayman Chemical, #706002), strictly following the manufacturers’ protocols. Malondialdehyde (MDA) levels were determined using a colorimetric assay kit (Sigma-Aldrich, #MAK085). Absorbances for catalase, SOD, and MDA were measured at 540 nm, 440 nm, and 532 nm, respectively.

### 3.6. Statistical Analysis

All experiments were performed with at least three independent biological replicates (n ≥ 3) to ensure reliability and account for biological variability. This sample size was considered sufficient for statistical power and is consistent with preclinical cell culture standards. Data are presented as mean ± standard deviation (SD). Statistical significance was determined using one-way or two-way analysis of variance (ANOVA), as appropriate, followed by Tukey’s honestly significant difference (HSD) test for multiple comparisons. A P-value less than 0.05 was considered statistically significant.

## 4. Results

### 4.1. Quercetin Suppressed the Viability of Gastric Cancer Cell Lines

The QCT significantly reduced the survival rates of AGS and MKN-45 GC cells in a dose- and time-dependent manner (P < 0.05; Figure S1, in the Supplementary File). The normal gastric GES-1 cell line exhibited higher IC_50_ values than the GC cell lines (Table S2, in the Supplementary File), indicating reduced sensitivity to QCT at the tested doses and durations. MTT assay results demonstrated that prolonged QCT treatment decreased IC_50_ values. Based on these findings, a 48-hour QCT treatment was selected for further studies.

### 4.2. Quercetin Modulated Hox Transcript Antisense Intergenic RNA and Downstream Target Gene Expression

**Figure 1.**
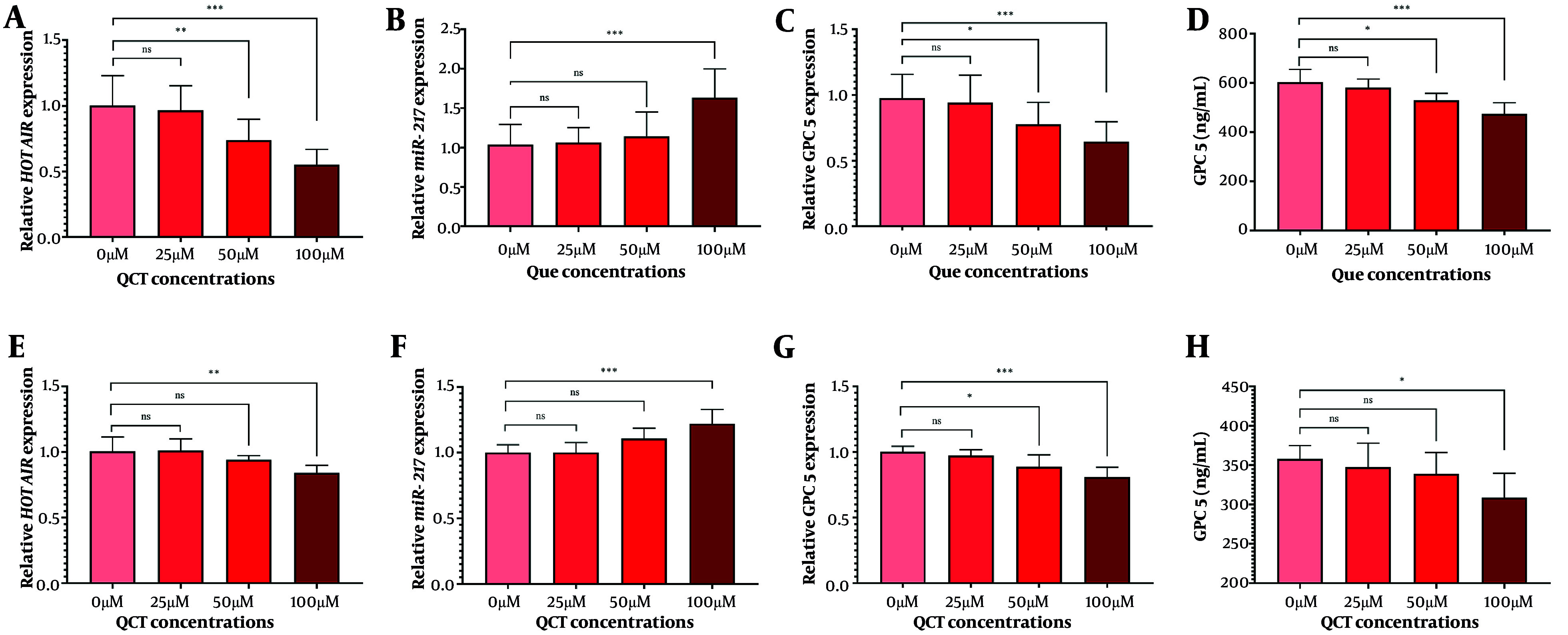
Quercetin (QCT) modified the expression of the hox transcript antisense intergenic RNA (HOTAIR)/microRNA-217 (miR-217)/glypican-5 (GPC5) axis. In the AGS cell line, QCT at 100 μM significantly decreased HOTAIR (A) and GPC5 (C) expression, while markedly increasing miR-217 (B). Similarly, QCT decreased HOTAIR (E) and GPC5 expression (G), and upregulated miR-217 (F) in the MKN-45 cell line. The GPC5 protein levels in both AGS (D) and MKN-45 (H) cell lines increased after QCT administration (*** P < 0.001, ** P < 0.01, and * P < 0.05; ns: Not significant; P < 0.05 was considered significant).

The QCT at 50 μM and 100 μM significantly reduced HOTAIR expression in both AGS and MKN-45 cells (P < 0.05). The GPC5 expression was also significantly decreased at these concentrations (P < 0.01). In AGS cells, 100 μM QCT increased miR-217 expression by 1.57-fold (P < 0.0001), while 25 μM and 50 μM did not yield significant changes (P > 0.05). In MKN-45 cells, 50 μM and 100 μM QCT induced 1.11-fold (P < 0.05) and 1.22-fold (P < 0.0001) increases in miR-217, respectively. The ELISA revealed that QCT significantly reduced GPC5 protein levels in both cell lines (P < 0.001) compared to controls (Figure 1). 

### 4.3. Quercetin Induced Apoptosis in Gastric Cancer Cell Lines

**Figure 2.**
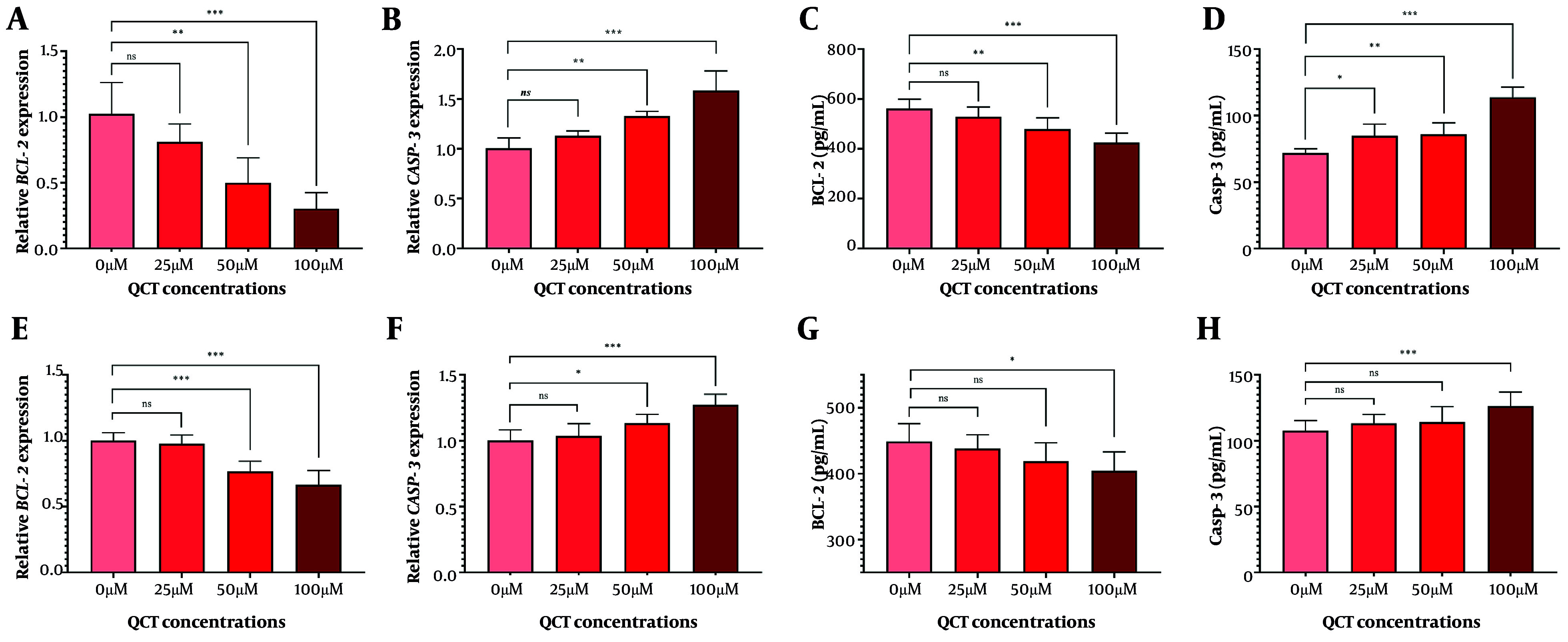
Quercetin (QCT) administration induced apoptosis. The QCT at 100 μM significantly downregulated BCL-2 levels (A and C), and upregulated CASP-3 (B and D) in the AGS cell line. In MKN-45 cells, QCT similarly decreased BCL-2 (E and G) and increased CASP-3 (F and H; *** P < 0.001, ** P < 0.01, and * P < 0.05; ns: Not significant; P < 0.05 was considered significant).

**Figure 3.**
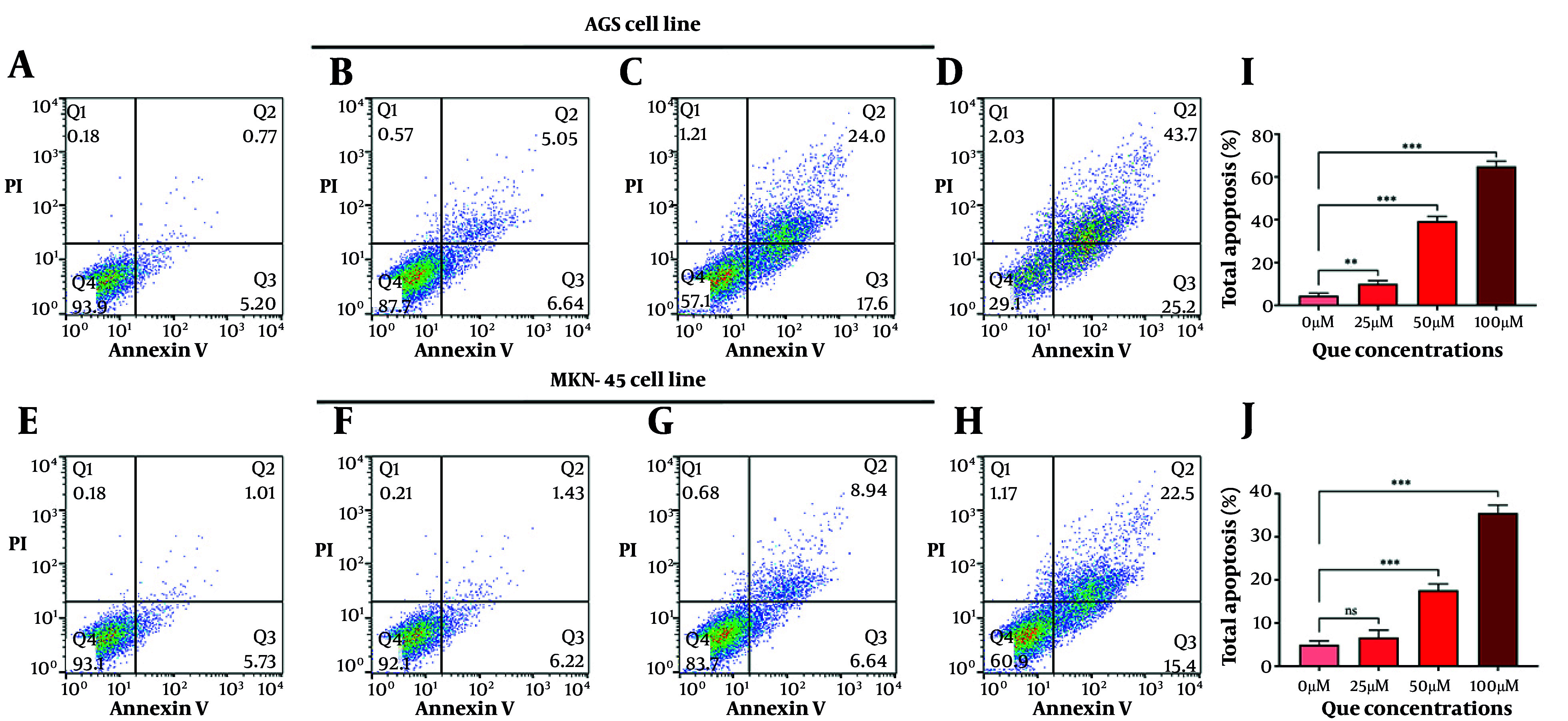
Quercetin (QCT) induced apoptosis in gastric cancer (GC) cell lines in a dose-dependent manner: Representative flow cytometry plots of annexin V-FITC/PI staining in AGS (A - D) and MKN-45 (E - H) cells treated with 0 μM (A, E), 25 μM (B, F), 50 μM (C, G), and 100 μM (D, H) QCT for 48 hours; quantitative analysis of total apoptosis (%) in AGS (I) and MKN-45 (J) cells (*** P < 0.001, ** P < 0.01, and * P < 0.05; ns: Not significant; P < 0.05 was considered significant).

The QCT at 50 μM and 100 μM markedly altered BCL-2 and CASP-3 gene and protein expression in AGS cells (P < 0.05). Similar modulation was observed in MKN-45 cells (Figure 2). Annexin V-FITC/PI staining demonstrated that QCT promoted dose-dependent apoptosis in AGS and MKN-45 cells (P < 0.05; Figure 3). In AGS cells, 25 μM, 50 μM, and 100 μM QCT increased total apoptosis by 2.96-fold (P = 0.002), 6.43-fold (P < 0.001), and 11.21-fold (P < 0.0001), respectively, compared to controls. In MKN-45 cells, these concentrations induced 1.65-fold (P = 0.172), 3.05-fold (P < 0.01), and 6.15-fold (P < 0.001) increases, respectively.

### 4.4. Quercetin Arrested the Cell Cycle in Gastric Cancer Cell Lines

**Figure 4.**
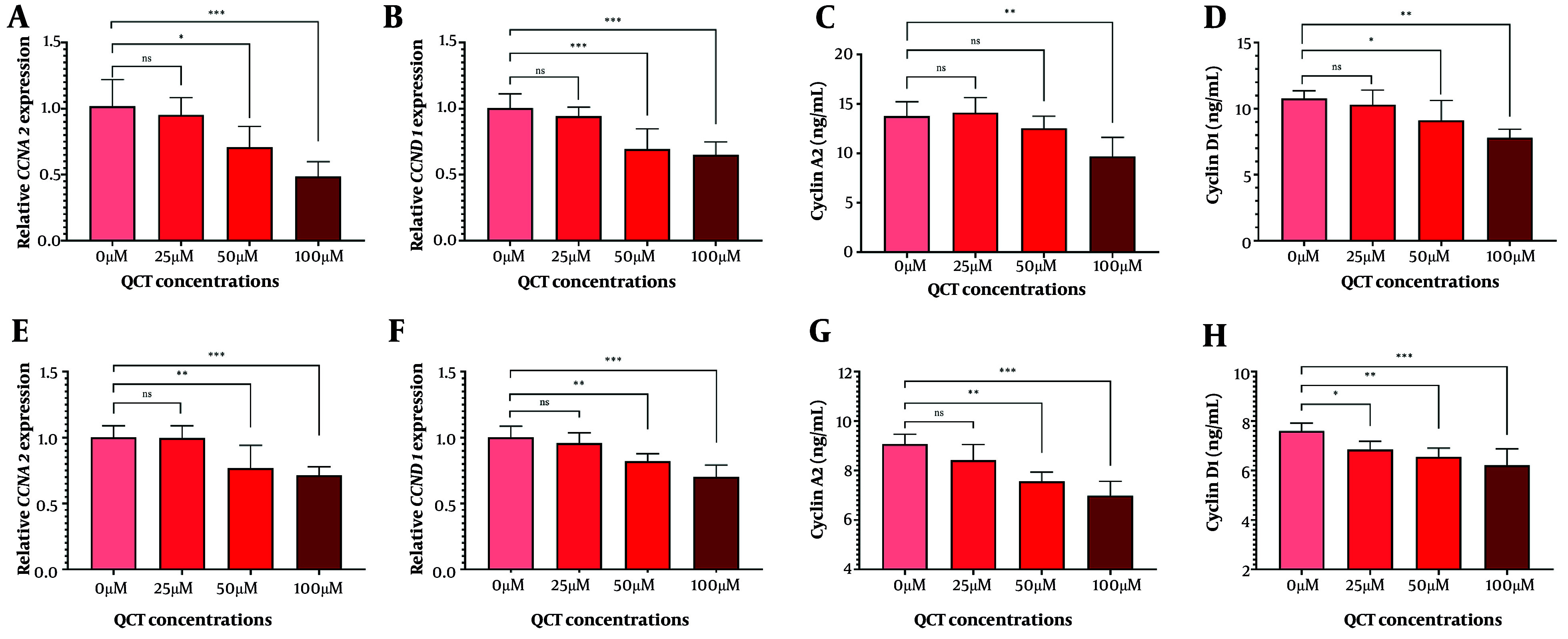
Quercetin (QCT) induced cell cycle arrest in gastric cancer (GC) cell lines. Expression of CCNA2 (A in AGS and E in MKN-45) and CCND1 (B in AGS and F in MKN-45), along with cyclin A2 (C in AGS and G in MKN-45) and cyclin D1 (D in AGS and H in MKN-45) protein levels was measured (***P < 0.001, ** P < 0.01, and * P < 0.05; ns: Not significant; P < 0.05 was considered significant).

In AGS cells, 50 μM and 100 μM QCT significantly reduced CCNA2 and CCND1 gene expression (P < 0.01). Cyclin D1 protein levels declined significantly at both doses, while cyclin A2 protein levels decreased significantly only at 100 μM (Figure 4). In MKN-45 cells, 100 μM QCT reduced cyclin A2 protein by 23.03% (P < 0.01) and gene expression by 28.72% (P < 0.001). Cyclin D1 protein decreased by 18.18% (P < 0.001), with a 30.07% reduction in CCND1 gene expression (P < 0.001).

### 4.5. Quercetin Suppressed Gastric Cancer Cell Invasion

**Figure 5.**
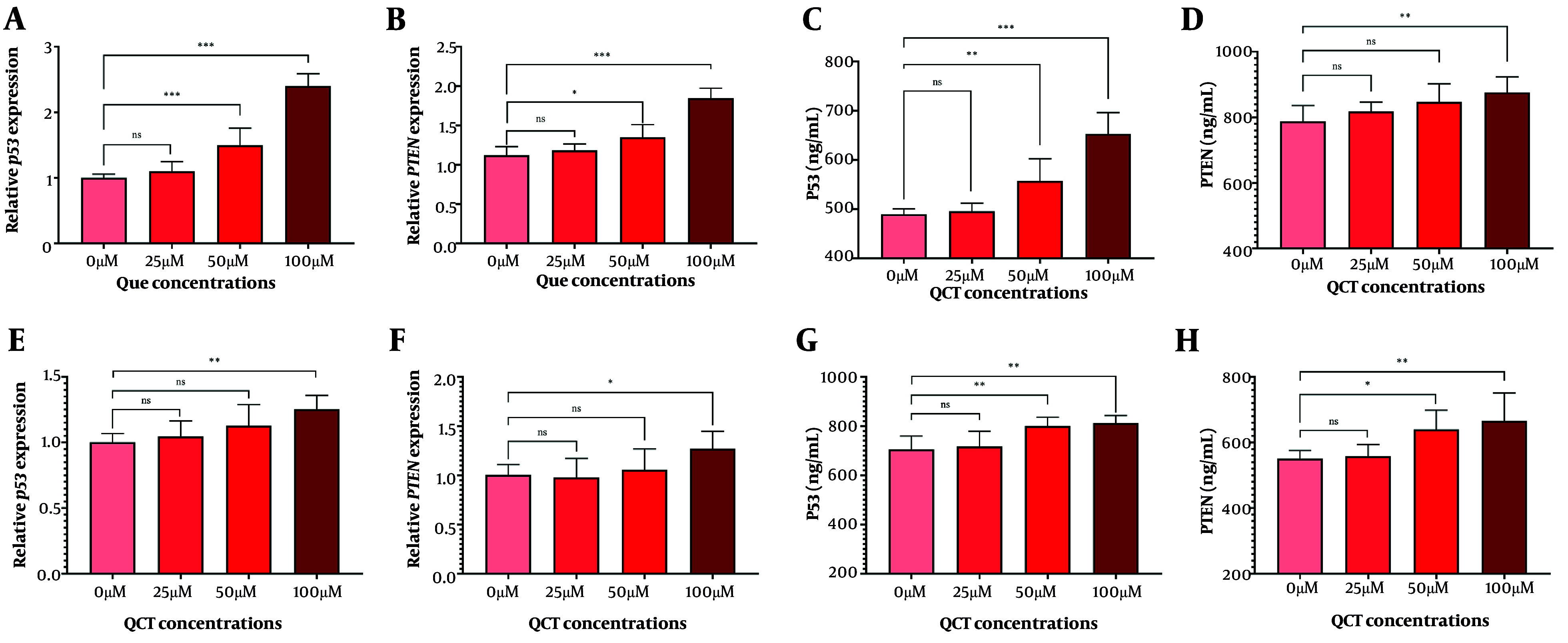
Tumor invasion was suppressed in gastric cancer (GC) cell lines after quercetin (QCT) treatment. Expression of p53 (A in AGS and E in MKN-45) and PTEN (B in AGS and F in MKN-45) genes increased upon QCT treatment. Additionally, 100 μM QCT significantly increased TP53 (C in AGS and G in MKN-45) and PTEN (D in AGS and H in MKN-45) protein levels (*** P < 0.001, ** P < 0.01, and * P < 0.05; ns: Not significant; P < 0.05 was considered significant).

The QCT at 50 μM and 100 μM increased TP53 and PTEN gene expression in AGS cells. TP53 protein levels rose by 1.14-fold (P = 0.005) and 1.34-fold (P < 0.0001), while PTEN increased by 1.11-fold (P < 0.001) at 100 μM. In MKN-45 cells, 100 μM QCT elevated TP53 and PTEN gene expression by 1.25-fold (P < 0.01) and 1.27-fold (P < 0.001), respectively, with protein analyses corroborating these findings (Figure 5). 

### 4.6. Quercetin Modulated Oxidative Stress and Lipid Peroxidation in Gastric Cancer Cell Lines

In AGS cells, 100 μM QCT significantly increased SOD and catalase activity (P < 0.0001), while 50 μM QCT significantly enhanced SOD activity (P < 0.05). No significant changes in MDA levels were observed in AGS cells at any QCT concentration (P > 0.05; Figure S2, in Supplementary File). In MKN-45 cells, 100 μM QCT increased SOD activity by 28.93% (P < 0.001), with moderate increases at lower doses. Catalase activity rose by 32.76% (P < 0.0001) at 100 μM, though increases at 50 μM and 25 μM were not statistically significant. The MDA levels decreased by 24.79% (P = 0.013) at 100 μM, with non-significant trends at lower concentrations.

## 5. Discussion

The QCT inhibited HOTAIR and GPC5 expression while upregulating miR-217 in a dose-dependent manner in both cell lines. Suppression was more pronounced in AGS than in MKN-45 cells, suggesting inherent differences in HOTAIR regulation — possibly due to baseline HOTAIR expression or molecular heterogeneity among GC subtypes (17). The GPC5 protein reduction was also less substantial in MKN-45 cells, potentially reflecting lineage-specific reliance on glypican-mediated pathways (18). These results align with evidence that GC cell lines possess unique molecular profiles affecting treatment responsiveness (19).

The HOTAIR is recognized as a key contributor to GC carcinogenesis, promoting malignancy by modulating intracellular pathways and altering microRNA levels (20, 21). The miR-217, sponged by HOTAIR, increases oncogene expression and epithelial-to-mesenchymal transition in GC cells. The antitumor role of miR-217 in gastrointestinal malignancies is largely mediated by inhibition of proliferation and invasion (11, 22). The GPC5, a target of miR-217, is implicated in tumor progression. Thus, HOTAIR, miR-217, and GPC5 are promising therapeutic targets, and agents modulating this axis represent potential strategies for GC management.

While this study elucidates the mechanistic action of QCT on the HOTAIR/miR-217/GPC5 axis, its potential interactions with other critical GC signaling networks, such as the transforming growth factor-beta (TGF-β) pathway, warrant further exploration (23). For example, QCT-mediated HOTAIR suppression may influence TGF-β effectors like SMAD proteins, as lncRNAs often serve as scaffolds for chromatin-modifying complexes. Additionally, the reduction in GPC5 may impact growth factor pathways such as vascular endothelial growth factor (VEGF) and fibroblast growth factor (FGF) signaling (24), potentially contributing to QCT’s anti-angiogenic and anti-invasive effects. Thus, QCT’s antitumoral actions likely involve both direct suppression of the HOTAIR/miR-217/GPC5 axis and indirect modulation of broader oncogenic networks.

The QCT-induced apoptosis and cell cycle arrest were dose-dependent in both cell lines, though MKN-45 displayed greater resistance. This may be due to differential expression of anti-apoptotic regulators or epigenetic modifications, as seen in chemoresistant GC models (22). Malignant cells evade apoptosis and proliferate excessively (25). Here, QCT at high doses significantly reduced cyclin A2 and D1 levels. Cell cycle modulation was more robust in AGS cells; MKN-45 cells required higher QCT concentrations for similar effects, possibly due to aberrant cyclin-dependent kinase activity in metastatic GC subtypes (26). Cyclin A2 upregulation drives proliferation, while its targeting induces arrest and apoptosis. Cyclin D1 is a GC progression marker, and its downregulation is therapeutically valuable (26).

Mutations in TP53 and loss of PTEN function are frequent in advanced GC (27, 28). In this study, QCT upregulated TP53 and PTEN, suppressing invasion in both cell lines, though MKN-45 exhibited greater PTEN protein variability at 100 μM. This reflects clinical observations of intra-tumor plasticity and compensatory signaling in GC (27). Excess reactive oxygen species and oxidative stress damage DNA and lipids, promoting angiogenesis and invasion (29). The QCT increased SOD and catalase activity, enzymes that convert reactive oxygen species into less reactive molecules. While both cell lines showed increased antioxidant enzyme activity, only MKN-45 exhibited significant MDA reduction, underscoring lineage-specific differences in lipid peroxidation dynamics and the need for diverse models when evaluating phytochemicals (22).

Despite compelling in vitro data, QCT’s clinical translation is challenged by poor bioavailability due to extensive first-pass metabolism and low water solubility (30, 31). Innovative delivery systems, such as nanoparticles and liposomes, are being developed to improve QCT’s bioavailability and targeted delivery. The mechanistic insights provided here support the rationale for future in vivo studies employing such strategies to assess QCT’s therapeutic efficacy.

Limitations include the exclusive use of in vitro models, which cannot fully capture the complexity of the tumor microenvironment or systemic pharmacology. The study focused narrowly on the HOTAIR/miR-217/GPC5 axis, not excluding other contributing pathways. Furthermore, the connection between TP53/PTEN upregulation and reduced invasion was correlative; genetic knockdown studies are needed for definitive causal inference. Future research should employ in vivo models and broader pathway analysis to facilitate translation of these promising results.

### 5.1. Conclusions

This study demonstrates that QCT exerts significant antitumor effects in GC by targeting the HOTAIR/miR-217/GPC5 pathway. AGS cells were more sensitive to QCT, with marked cyclin downregulation and strong apoptotic activation, while MKN-45 cells displayed diminished responses, highlighting the impact of molecular heterogeneity on therapeutic efficacy. The QCT further inhibited invasion via TP53/PTEN upregulation and modulated oxidative stress by enhancing antioxidant enzyme activity and reducing lipid peroxidation in MKN-45 cells. These preclinical in vitro findings reveal the diverse mechanisms through which QCT suppresses GC cell proliferation and survival, suggesting its potential for further development as an adjunctive therapy. However, these findings are preliminary and require validation in complex in vivo models. Variations in lineage-specific efficacy emphasize the need for rigorous preclinical studies in animal models before clinical trial consideration. Future research should refine dosing strategies and explore combination therapies to address resistance mechanisms.

## supplementary material

ijpr-24-1-165480.pdf

## Data Availability

The dataset presented in the study is available on request from the corresponding author during submission or after publication.
